# Gd-EOB-DTPA dynamic contrast-enhanced magnetic resonance imaging is more effective than enhanced 64-slice CT for the detection of small lesions in patients with hepatocellular carcinoma: Erratum

**DOI:** 10.1097/MD.0000000000014217

**Published:** 2019-01-11

In the article, “Gd-EOB-DTPA dynamic contrast-enhanced magnetic resonance imaging is more effective than enhanced 64-slice CT for the detection of small lesions in patients with hepatocellular carcinoma”,^[1]^ which appeared in Volume 97, Issue 52 of *Medicine*, the authors would like to note they also received funding from Natural Science Foundation of Guangxi (2015GXNSFFA139004, 2016JJB140027).
